# A Boolean Model of the Cardiac Gene Regulatory Network Determining First and Second Heart Field Identity

**DOI:** 10.1371/journal.pone.0046798

**Published:** 2012-10-02

**Authors:** Franziska Herrmann, Alexander Groß, Dao Zhou, Hans A. Kestler, Michael Kühl

**Affiliations:** 1 Research Group Bioinformatics and Systems Biology, Institute for Neural Information Processing, Ulm University, Ulm, Germany; 2 Institute for Biochemistry and Molecular Biology, Ulm University, Ulm, Germany; 3 International Graduate School in Molecular Medicine, Ulm University, Ulm, Germany; Northwestern University, United States of America

## Abstract

Two types of distinct cardiac progenitor cell populations can be identified during early heart development: the first heart field (FHF) and second heart field (SHF) lineage that later form the mature heart. They can be characterized by differential expression of transcription and signaling factors. These regulatory factors influence each other forming a gene regulatory network. Here, we present a core gene regulatory network for early cardiac development based on published temporal and spatial expression data of genes and their interactions. This gene regulatory network was implemented in a Boolean computational model. Simulations reveal stable states within the network model, which correspond to the regulatory states of the FHF and the SHF lineages. Furthermore, we are able to reproduce the expected temporal expression patterns of early cardiac factors mimicking developmental progression. Additionally, simulations of knock-down experiments within our model resemble published phenotypes of mutant mice. Consequently, this gene regulatory network retraces the early steps and requirements of cardiogenic mesoderm determination in a way appropriate to enhance the understanding of heart development.

## Introduction

The heart is the first functional organ to develop in mammals. After the end of gastrulation, cardiogenic progenitor cells constitute the cardiac crescent in the anterior mesoderm of the murine embryo. At this stage the cardiogenic mesoderm splits from a common cardiovascular progenitor cell population [1], [2] into two areas of differential gene expression: the so-called first heart field (FHF) and the second heart field (SHF). Cells of the FHF build the primary heart tube and later mainly contribute to the left ventricle, most of the atria and provide a minority of cells of the right ventricle. Cells of the SHF mainly contribute to the right ventricle, the outflow tract and the atria [3], [4].

Underlying regulatory factors control these differentiation processes. The induction of mesoderm depends on canonical Wnt signaling [5]. After mesoderm formation cardiogenic precursor cells are characterized by the expression of the transcription factor Mesp1 [6]. Endodermal signals such as Bmp2 were also described as being crucial for cardiogenesis [7], [8], [9]. These signals activate a variety of transcription factors of the cardiogenic mesoderm like Nkx2.5 or GATA factors [7], [8], [10]. Some of the cardiac transcription factors can be assigned to one of the two heart fields. The transcription factors Isl1, Foxc1/2, Tbx1 and the ligand Fgf8 determine the area of the SHF, while the transcription factor Tbx5 is only expressed in the FHF [11], [12], [13], [14]. It is thought that intrinsic wiring among these cardiac factors determines the progression of cardiac differentiation and the division into subdomains of differential gene expression. Heart development can severely be impaired in case a regulatory factor of cardiogenesis is missing. Several studies analyzed specific interactions within gene regulation of early mammalian heart development using knock out or knock down approaches of individual factors. A deeper understanding of the cardiac gene regulatory network requires the implementation of this network as a computational model and its subsequent analysis by computational simulations.

Expression of a gene is regulated by input signals given by transcription factors binding to the regulatory region of the gene. The strength of transcription, e.g. the amount of primary transcript, can be depicted as a function depending on the concentration of these regulatory transcription factors. This function often follows a sigmoidal behaviour, which is governed by cooperativity in a first stage and controlled by saturation at later stages resulting in a switch-like behavior. This property ensures defined levels of gene expression for a wide range of concentration levels. This sigmoidal function of gene expression can be approximated as a step function [15]. A common approximation of the possible states of a gene is therefore to consider a gene to be active or inactive [15]. These two states of a gene correspond to a present and to an absent gene product and can be encoded as Boolean logical values: true (1) and false (0). Dependencies between genes, e.g. whether a transcription factor acts as a transcriptional activator, repressor or both, can then be captured by Boolean functions which map the state of a gene regulatory network to a succeeding state. These functions allow a Boolean model to exhibit dynamical behavior in simulations. Boolean logic network models have been used to model e.g. endomesodermal territories in the sea urchin [16], the hrp regulon of *Pseudomonas syringae* [17], and the bistable lac operon in *E. coli* [18]. Every Boolean model has a finite number of states, as the state of each gene is represented by one of two possible values. For k genes this results in 2^k^ possible state combinations. It follows, that starting a simulation from an initial state and following synchronous state transitions according to the Boolean functions, the model eventually ends up in recurring states, a cycle. A degenerated cycle may consist of a single Boolean state. These recurring states are called attractors. They can correspond to observed expression profiles or phenotypes in gene regulatory networks [19], [20], [21].

We here introduce a Boolean model for the early cardiac gene regulatory network of the mouse, containing known core genes required for cardiac development and FHF/SHF determination. The model is based on published temporal and spatial expression patterns of relevant transcription factors and growth factors and includes known regulatory interactions taking into account whether a transcription factor acts as an activator or repressor on a given target gene. We are able to show that this computational model is able to reproduce different states observed during cardiac development. Model simulations demonstrate that stable states of gene expression representing either the FHF or the SHF are encoded within the wiring of gene interactions. Thereby, we provide an insight into the functional properties of the cardiac gene regulatory network. This will be an important basis for further enlargements of the network and for *in-silico* predictions of genetic interactions.

## Results

### A Gene Regulatory Network for Early Murine Cardiogenesis

**Figure 1:**
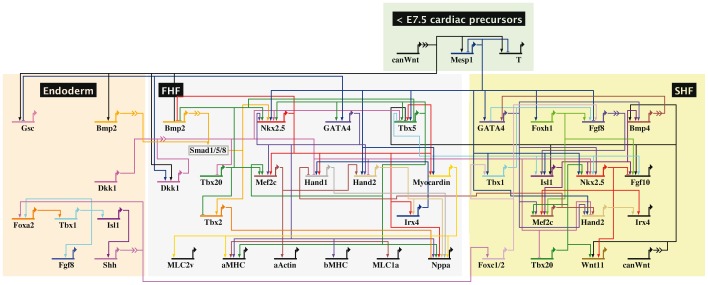
Gene regulatory network during early murine cardiac development. The overview comprises published gene regulations in early heart precursor cells, focussing on two areas with different gene expression, the first heart field (FHF) and the second heart field (SHF). A differentiation of the two heart fields happens around E7.5. Signaling of the endoderm which influences cardiac progenitors was included in this overview as well as early mesodermal signals. Genes are represented by their regulatory region and their transcriptional start site. Information from other genes is processed within the regulatory region. The transcriptional start site of a gene indicates expression and influences gene transcription of other genes. Arrow heads indicate activation and bar heads inhibition of gene transcription. Broken lines represent intercellular signaling with an integrated signal transduction cascade.

For constructing a gene regulatory network of early cardiac development we collected published data. An overview of cardiac genes and their interactions is provided in Figure 1 and Table S1. The expression of genes and their interactions take place in a temporal and spatial frame, as marked by colored boxes in Figure 1. The network is characterized by early signaling events during gastrulation resulting in cardiac specification and subsequent signaling activities at the cardiac crescent stage which separate the cardiac progenitor cell population into the territories of the FHF and the SHF lineages. Furthermore, signaling from the endoderm was also included into our model.

Early canonical Wnt signaling is required for the activation of the pan-mesodermal transcription factor Brachyury [5], [22] and for the cardiac specific expression of the transcription factor Mesp1 [10]. Mesp1 subsequently activates various cardiac factors, initiating the cardiac crescent stage. Mesp1 upregulates the expression of genes of both heart fields, e.g. *Nkx2.5, GATA4, Tbx1* and *Tbx5* [8], [10]. At the same time, Mesp1 inhibits expression of genes of other developmental fates (not included into the model), e.g. endodermal genes and the mesodermal gene *Brachyury* which later supports posterior mesoderm formation and axial development [10], [23], [24]. In the FHF, cardiac transcription factors like Nkx2.5, GATAs and Tbx5 build an intertwined positive feedback circuit to stabilize their expression. They activate downstream regulatory genes like *Hand* genes or *myocardin*. Finally, regulatory factors upregulate a set of differentiation genes. Genes being specific for terminal differentiation such as *myosin light chain* genes *(MLC)* or *myosin heavy chain* genes *(MHC)* code for structural proteins and constitute the end of the developmental processes described by the regulatory network (Figure 1, FHF area). Similarly, a network of molecular interactions exists in the SHF (Figure 1, SHF area). Furthermore, the endoderm has been shown to influence cardiogenesis, especially by the signaling factors Bmp2 and Dkk1. In addition, in the heart looping stage the regulatory factors Isl1, Tbx1 and Fgf8 are also expressed in the pharyngeal endoderm. There, Shh activates Tbx1 through the Forkhead transcription factor Foxa2. Shh might also signal to the SHF and regulate gene expression during later stages [12].

### A Boolean Model of the Cardiac Regulatory Network

**Figure 2:**
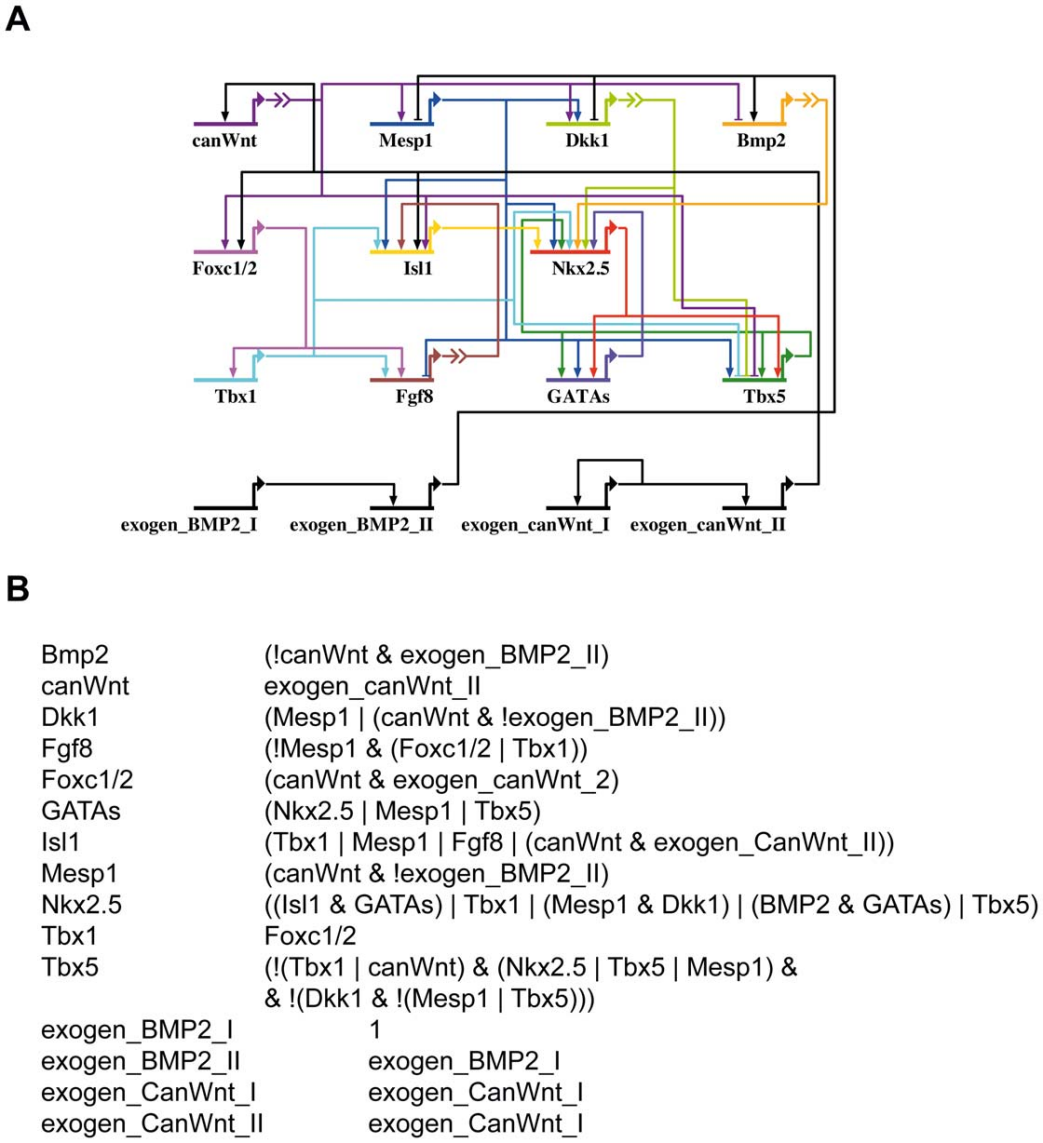
Boolean model of the cardiac gene regulatory network. (A) Only genes included into the model and their regulations are shown. Regulations are based on published data. (B) Boolean transition functions of the network in (A). All genes of the network are listed on the left side. A new state for each gene is derived from the value of the Boolean function on the right side based on the preceding states of genes. Input variables are combined by Boolean functions: ! = NO, | = OR or & = AND. Brackets determine the order of evaluation, starting with the innermost. Exogen BMP2 I+II: inputs for non cardiac BMP2 signaling; exogen CanWnt I+II: inputs for non cardiac canonical Wnt signaling.

We implemented the core cardiac genes and their interactions (Fig. 2A) as a Boolean model. The interactions of the involved genes and signaling factors were gained from published data and are enlisted in detail in Table S2. The corresponding Boolean functions are given in Figure 2B. This computational model represents the core regulatory interactions of the gene regulatory network of cardiogenesis as presented in Figure 1.

As introduced, cardiac development also depends on signals derived at particular time points of development from other tissues such as the endoderm and thus are not included in the core cardiac gene regulory network. Those are represented in our model by four genes: *exogen BMP2 I, exogen BMP2 II, exogen CanWnt I, exogen CanWnt II*. Bmp2 signaling for example from the endoderm is important for the induction of cardiogenic mesoderm. In order to represent the required activation of Bmp2 at the correct time point, the cardiac progenitor cell state, we modeled a temporal delay by the two genes exogen BMP2 I and exogen BMP2 II. Similarly, exogen CanWnt I and exogen CanWnt II are used to activate canonical Wnt signaling at the cardiac crescent state in the SHF. This represents the described canonical Wnt signaling in the SHF at E7.5 [25].

### Expectations of the Boolean Model

**Figure 3:**
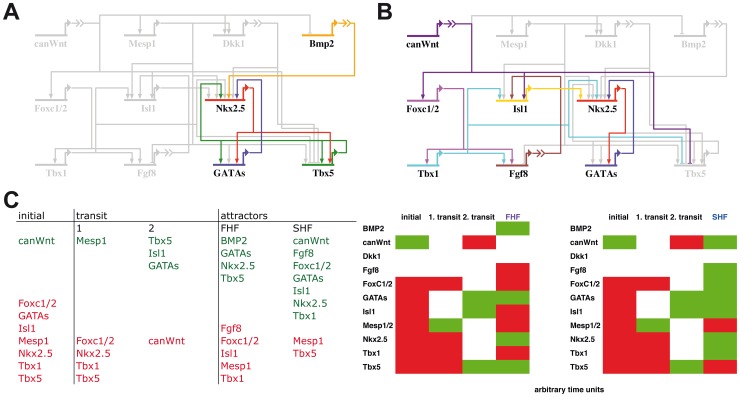
Expected network states. Literature derived state descriptions are expected to appear in simulations, presented by a defined set of genes to be active. In (A) a state defined as FHF state with the according gene activity and in (B) the expected SHF state are presented. Colored genes are active, while gray genes are inactive. In (C) states are listed which are expected to appear during a simulation course. In the initial state of the network which corresponds to early mesoderm development, only canonical Wnt signaling is expected to be present, while genes for differentiation of cardiogenic precursor cells are still inactive. A simulation of the network model is expected to pass transition steps one and two. These correspond to transient states of early cardiogenic mesoderm expressing Mesp1 and to the common cardiac progenitor cell population. Finally, the simulation is expected to result in either the FHF or the SHF state. Genes which are expected to be expressed at a certain state are listed in green, and genes which should not appear in a state are shown red. On the right side, the same information as in the table on the left is shown in a manner similar to the results in Figures 4 and 5. In this table, white color indicates that no expectation for the gene activity is specified.

Simulations of the computational Boolean model are expected to reproduce gene expression profiles as closely as possible and in the same temporal sequence as they appear during cardiogenesis *in vivo*. Therefore, the temporal and spatial expression patterns of genes included in the model were collected from publications (Table S3). According to the collected data, expected attractors representing the FHF and SHF as well as the expectations for transient states were defined (Figure 3). Furthermore, as genes are stably expressed in a certain area the model should not only reproduce a gene expression profile, but exhibit also stability of these states. Thus, we expect the expression profiles of the FHF and of the SHF to be represented in attractors of the network model.

**Figure 4:**
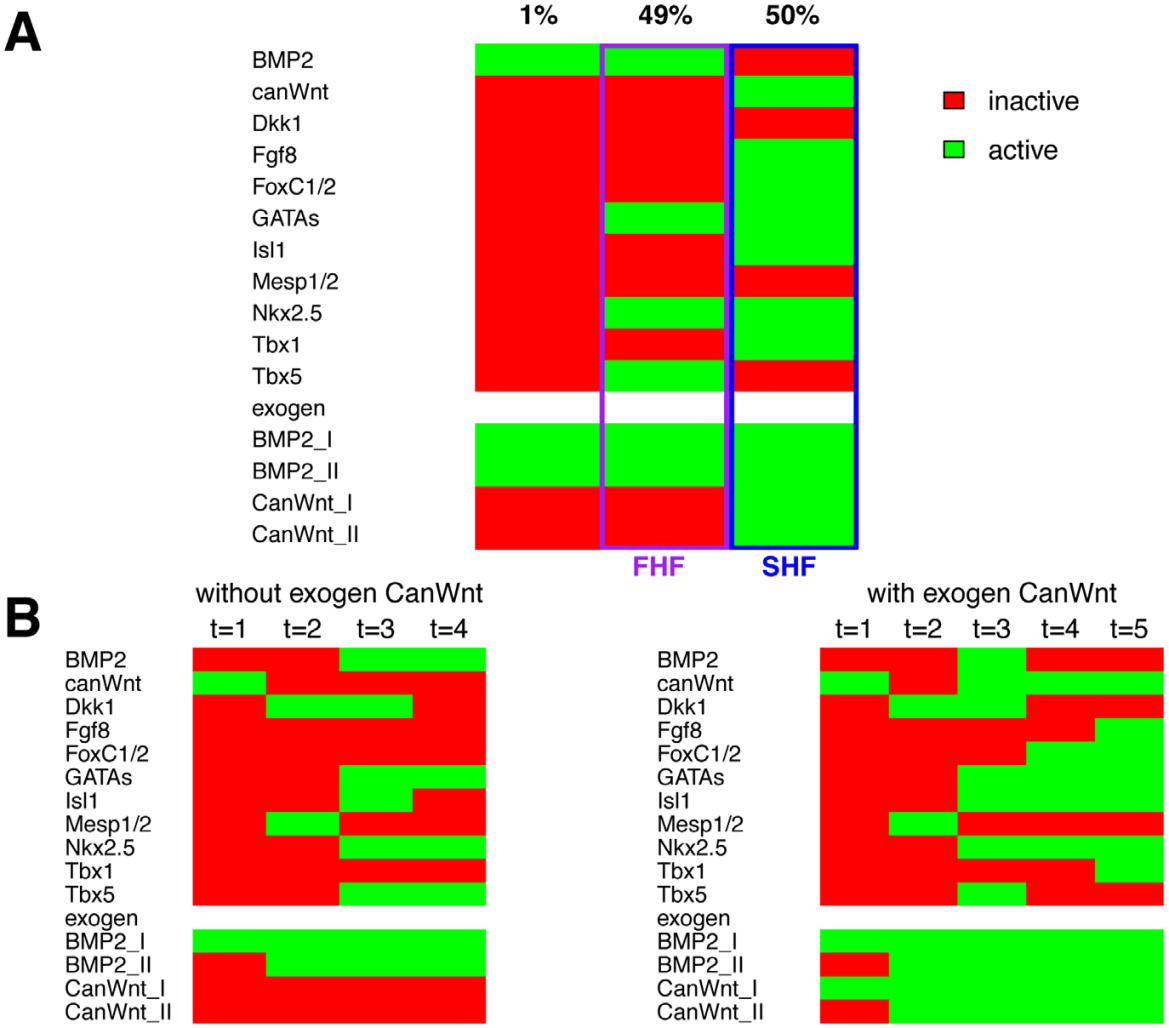
Results of network model simulations. Gene activity for all genes of the model is presented at distinct network states. A green box indicates activation whereas a red box denotes inactivation of a gene. (A) Summary of the analysis starting simulations from all possible initial states. All runs resulted in one of the three attractors shown. 49% of the simulations resulted in an attractor for the FHF (indicated in purple), 50% in an attractor mimicking the SHF state (indicated in blue) and 1% of the simulations yield an attractor without activation of core cardiac genes. (B) Simulation of time courses from expected initial states of the cardiac gene regulatory network model. Initial state setups differ only in the activation of canonical Wnt signaling at t = 3 (with exogen CanWnt I or without exogen CanWnt II). State transitions match expectations of intermediate state expressions and end in the attractors for FHF and SHF lineages.

**Figure 5:**
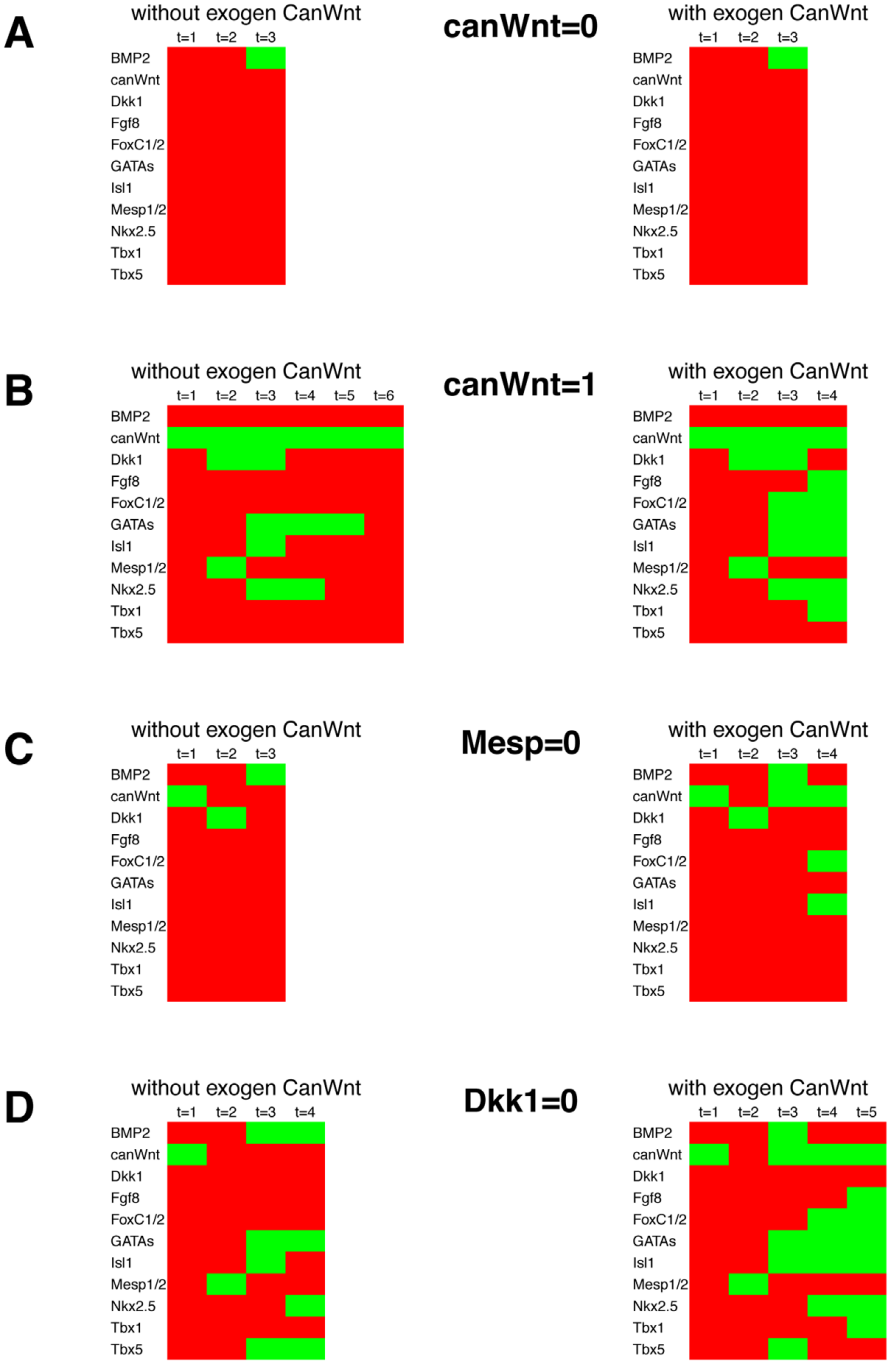
Knock-down or overexpression simulations of canonical Wnt signaling, Mesp1 and Dkk1. For knock-down or overexpression a gene is set to 0 or 1, respectively, throughout the simulation. Apart from that, the simulation is performed as in Figure 4B, starting from the defined initial state and receiving signals as in the setup without non-cardiac canonical Wnt signaling or as in the setup with non-cardiac canonical Wnt signaling. (A) Canonical Wnt signaling is switched off (canWnt = 0). This leads in both setups to attractors lacking active cardiac genes. (B) Upon canonical Wnt overexpression (canWnt = 1), no FHF but a SHF forms. (C) If Mesp1 is switched off (Mesp1 = 0), no FHF builds. In the SHF setup, major cardiac regulator genes are inactive after three state transitions. (D) Upon Dkk1 knock-out, attractors and transitions states stay the same. Activation of Nkx2.5 is delayed by one step compared to the undisturbed simulation.

The expected gene expression profiles for a FHF attractor and for a SHF attractor are given in Figure 3A and Figure 3B, respectively. In the FHF the genes *Bmp2*, *Nkx2.5*, *Gatas* and *Tbx5* are expected to be active, while genes which usually only appear in the SHF are inactive. In the SHF *canWnt*, *Tbx1*, *Fgf8*, *Foxc1/2*, *Gatas, Nkx2.5* and *Isl1* are active, while *Tbx5* is expected to be inactive. Figure 3C summarizes the initial states, transition states and the attractors of the FHF and the SHF.

### The Network Model Exhibits Stable States for the FHF and the SHF and Reproduces Temporal Development

To analyze the cardiac network, we simulated all possible initial state combinations (2^15^ = 32768 states for all genes) with the cardiac network model. This analysis detects all possible Boolean attractors of the Boolean model (Figure 4A). Our simulations lead to three attractors. One attractor appears only for 1% of the initial states and contains no active cardiac genes. Another attractor appears in 49% of the cases. In this state the genes *Bmp2, Tbx5, GATA* and *Nkx2.5* are active, while other genes are inactive. This attractor resembles the gene expression in FHF cells (compare with Figure 3A) and will be called the FHF attractor from now on. An additional attractor appears for 50% of all initial states and corresponds to the SHF gene expression state (as specified in Figure 3B). We call this the SHF attractor. This finding indicates that the wiring of the network determines gene expression in the first and second heart field.

Next, we analyzed the temporal sequence of network states leading to the FHF and the SHF attractors. For this purpose we included relevant biological information to define an appropriate initial setting of the network. At the beginning of gastrulation, canonical Wnt signaling is active and initiates the determination of the mesodermal and cardiac cell lineage [26]. None of the other cardiac specific genes included in our network are active at that time. Therefore, we defined an initial state in which only canonical Wnt signaling is active for further simulations with our network model (compare expectation for the initial state in Figure 3C). Furthermore, both heart fields are induced by endodermal Bmp signaling after the induction of mesoderm. We modeled this by the external signals exogen BMP2 I and exogen BMP2 II which initiate Bmp signaling at the appropriate time point.

The initial state with active cardiac canWnt and exogen (endodermal) BMP2 leads to a FHF attractor (Figure 4B, left panel). After gastrulation, the SHF receives additional canonical Wnt signaling while the FHF is unaffected. Therefore, we additionally specified this external signal by exogen CanWnt I and II for the setup which re-activates canonical Wnt signaling at the cardiac crescent state. The initial state with active canWnt, exogen BMP2 and exogen CanWnt leads to a SHF attractor (Figure 4B, right panel).

Setups giving rise to both FHF and SHF contain an initial state with active canWnt signaling and active exogen BMP2 I. The difference between both setups is the additional activation of exogen CanWnt I in the setup leading to the SHF. The use of external signals allows us to compare the intrinsic states of the network during the simulations to a temporal developmental process in the developing organism, which integrates signals from non-cardiac tissues.

The state transitions of the network, which occur during the simulation, can be compared to a temporal process in the developing organism. From the initial state on (marked by time point t = 1) we follow the state transitions towards the final attractors for the two setups differing in exogen CanWnt I activation (Figure 4B). There are three state transitions leading to the FHF attractor. Following an active canonical Wnt signaling Mesp1 and Dkk1 are expressed at time point t = 2. Transient Mesp1 expression is described in cardiac precursor cells during gastrulation [6], [27], [28]. Mesp1 activates a variety of cardiac regulatory factors [8], [29]. The network state at t = 2 of the simulation resembles the gastrulation stage *in vivo* (compare transit 1 in Figure 3C). Cardiac regulatory genes *Nkx2.5, GATAs, Isl1* and *Tbx5* are activated at t = 3 of the simulation. Active genes of this state resemble the expression in the common cardiovascular progenitor cell population where Isl1, Tbx5, GATAs and Nkx2.5 are present [1], [2], [30]. The expected expression for this state is defined in transition state 2 in Figure 3C. After one additional transition the FHF resembling attractor appears (Figure 4B, without exogen CanWnt I, t = 4).

In the setup with active exogen CanWnt I, cardiac canonical Wnt signaling is reactivated at the state of the common cardiovascular progenitor cell (Figure 4B, with exogen CanWnt I, t = 3). States leading to the common cardiovascular progenitors are identical in comparison to the setup without exogen CanWnt I. Canonical Wnt signaling leads to the activation of SHF genes and thereby to the inhibition of the FHF transcription factor Tbx5. The SHF attractor is constituted at time points t = 4 and t = 5. In summary, the tracing of state transitions in the simple Boolean model substantiates the literature derived expectations.

To verify that canonical Wnt signaling is the signaling event that drives cells towards a SHF fate, we eliminated the influence of exogen CanWnt II in the model by keeping exogen CanWnt II = 0 throughout the simulation. This is in line with investigations by Klaus et al. [31] and Lin et al. [25] which showed that Wnt/â-catenin dependent transcription is upregulated at E7.5 in the second heart field. For both initial setups the network attains a FHF attractor, passing the same transition states as for the wildtype without exogen canWnt (Figure 4B, left side). It demonstrates that in our network model cardiac canonical Wnt signaling which is induced from non cardiac tissue is sufficient to establish the SHF attractor.

### The Boolean Model Reproduces Knock-out and Overexpression Phenotypes

For most of the genes involved in the presented cardiac network, knock-out mice have been studied with respect to cardiac development. To analyze whether our model will produce attractors comparable to knock-out phenotypes, the corresponding gene was kept off at all time points of simulation.

Without Wnt signaling no mesoderm is formed, which is the prerequisite for cardiogenesis [5]. For this, we set canWnt = 0, simulating the ablation of cardiac canonical Wnt signaling (Figure 5A). In the simulations of both setups Mesp1 is not activated resulting in absence of cardiac mesoderm at time t = 2 and thus further cardiac factors are not activated. The predictions of the model simulations are consistent with knock-out experiments in mice, where upon ablation of canonical Wnt signaling no mesoderm forms [5]. We also simulated overexpression of cardiac canonical Wnt signaling by constantly activating Wnt signaling (canWnt = 1) (Figure 5B). It is known that a sequence of different levels of canonical Wnt signaling are required for proper heart development [26]. In both initial setups, mesoderm is induced upon Wnt overexpression as indicated by Mesp1 activation (Figure 5B, t = 2). In the setup without exogen canonical Wnt signaling, some of the cardiac genes are activated after expression of Mesp1, but no defined heart field is established, leading to an attractor without activated cardiac genes. In contrast, starting by the setup with non-cardiac canonical Wnt signaling, the simulation soon reaches the SHF attractor. In our model a FHF attractor is not formed, while the SHF attractor is reached one time step earlier. Conditional overexpression of β-catenin (a key transducer of canonical Wnt signaling) disrupts primary heart tube formation, a FHF derived structure and leads to an expanded Isl1 expression in the SHF [25], [31].

The mesodermal gene *Mesp1* induces the expression of many cardiac genes. In the mouse embryo Mesp2 can compensate for some Mesp1 functions. Since Mesp2 is not an explicit part of the network model, but merely is represented by Mesp1, model simulations switching off Mesp1 (Mesp1 = 0) resembles a double knock-out of Mesp1 and Mesp2 in the mouse embryo [28]. These double knock-out mutant embryos do not form cardiac mesoderm. In our model, switching of Mesp1 leads for the setup without exogen canonical Wnt signaling to an attractor without any expression of cardiac genes. For the setup with exogen CanWnt signaling, no cardiac genes are active in the appropriate time frame when a common cardiac progenitor cell should be established (Figure 5C, with exogen CanWnt I, t = 3). If the time window for differentiation of the cells passes, some other fate will be adopted. In our simulations, the network reaches the SHF attractor after a number of further state transitions due to a lack of genes for alternative fates and a lack of further signaling input from other tissues. Since our model does not exhibit cardiac gene expression within two time steps past the gastrulation state, it is in accordance with the reported *in vivo* results.

Dkk1 has been demonstrated to induce cardiac marker genes together with Mesp1. Without Mesp1, Dkk1 inhibits their expression [8]. The knock-out simulation of Dkk1 in the presented cardiac gene regulatory network model exhibited a delay of Nkx2.5 activation by one transition state. Besides this, the attractors and state transitions are are identical in comparison to the undisturbed network (Figure 5D). This simulation result demonstrates that in our model Dkk1 is not important for the correct specification and differentiation of cardiac mesoderm into FHF and SHF lineages. As shown in [32], Dkk1 and Dkk2 double mutant mice have no immediate effect on heart development. Later, these double mutant mice exhibit defects in proliferation and hypertrophy in the heart. These defects affect the heart later than it is displayed in our network model and may result from a later impact of Dkks on heart development. Furthermore, genes regulating proliferation are not integrated into the network model and can not be detected in our simulations.

The consistency between the biological phenotypes described in the literature and our corresponding simulations demonstrate that our Boolean model closely incorporates the molecular mechanisms underlying cardiac development as they have been investigated so far.

## Discussion

In the presented model of the cardiac gene regulatory network we collected and integrated knowledge about major regulatory factors required for heart development and their interactions. The construction of a Boolean model of the cardiac regulatory network allowed us to show that the interactions within the network lead to stable regulatory states representing the FHF and SHF lineages at E7.5 of murine development and indicates a robustness within the network wiring.

### The Boolean Model Describes Early Cardiac Development

We show that the computational model presented here is sufficient to describe basic regulations of early heart development in mice. Simulations of this model reproduce temporal behavior of heart developmental processes reflecting important stages as the canonical Wnt expression phases [26], a common cardiac progenitor cell stage [1], [2] and the FHF and SHF phenotypes [3]. The model furthermore predicts the behaviour of the network upon particular disturbance. Switching off initial canonical Wnt signaling for example (Figure 5A; Wnt = 0) leads into a „no heart field“ attractor. This simulation result is consistent with the complete ablation of β-catenin in mice, when mesoderm is not formed [5]. Moreover, the transcription factor Mesp1 marks cardiac progenitor cells. Upon switching off Mesp1 in our model, either no heart field is developed or after activation of some cardiac factors and six state transitions a SHF phenotype is reached. After gastrulation, at about t = 3 or t = 4 cardiac genes are not expressed in the simulation. At this stage usually a cardiac fate is established. As Mesp1 expression, which is required for cardiogenic induction, is not present and further network wiring which could direct the network towards another attractor were not included in the network the simulation ends in a SHF attractor. Nevertheless, these results indicate that cardiac formation is impaired upon Mesp1 ablation. This corresponds to the finding that Mesp1 ablation in mouse leads to severe defects of the formation of cardiogenic mesoderm. Upon ablation of Mesp1 and Mesp2 no cardiac mesoderm is detected at all [27], [28]. Furthermore, Dkk1 knock-out in our network model revealed no defect in differentiation towards FHF and SHF fates and has barely an impact on the activation of other cardiac factors. This is comparable to Dkk1/Dkk2 double mutant mice, which do not show defects in heart fate decision at the early stages of development which the presented cardiac gene regulatory network model covers [32]. Therefore, known in-vivo knock-out experiments can be reproduced with the here presented mathematical network model.

The stable state of the FHF is an example of intertwined positive feedback regulation, as the factors involved activate each otheṙs expression. The stabilization of the SHF is less clear. In our computational model, it is mediated by canonical Wnt signaling. Canonical Wnt signaling has been shown to act in the SHF from E7.5 [25]. A conditionally ablated β-catenin (a mediator of canonical Wnt signaling) mutant mouse leads to shortened SHF derived right ventricle and outflow tract. Furthermore, SHF specific gene expression, especially Isl1, is diminished in the outflow tract and in the splanchnic mesoderm [25], [31]. Analyses of a Mesp1-induced gain-of-function mutant of β-catenin [31] shows that the formation of the linear heart tube (usually promoted by FHF cells) was disrupted and the expression of Isl1 was expanded. This observation is in agreement with the overexpression study in our Boolean model (Figure 5B; Wnt = 1) which lacks the formation of a FHF state. Together, these results indicate that canonical Wnt signaling is a major regulator of SHF identity.

### Wiring within the Network Results in Robustness

Our analyses demonstrate an important property of the network wiring: robustness. Robustness ensures that aberrations in the temporal appearance or in amounts of transcription factor expression between cells do not alter the cell’s next stable regulatory state.

Our simulations show that there are stable states of the network, which resemble the gene expression patterns of the FHF and SHF, respectively. This analysis investigated all possible initial states and all transitions towards the attractors. The simulation of all possible initial states has limited biological relevance, because most of these initial states will not appear in a mouse embryo *in vivo*. Nevertheless, the experiment shows that these attractors are an intrinsic property of the Boolean network. The results can serve as a measure of the stability against random fluctuations in gene expression. Thus, most possible aberrations in states will eventually lead to a FHF or SHF attractor through a self-stabilizing mechanism contained in the wiring of the Boolean network.

Afterwards, for the progression of differentiation to a succeeding stable regulatory state this robustness needs to be overruled e.g. by a signal from external tissue. This signal can be transmitted by signaling ligands for example. The cardiac network model e.g. contains canonical Wnt signaling starting to signal from non-cardiac tissue in a defined time frame. This signal affects the target cells to arrive at a stable state, resembling the SHF. This state is only stable as long as canonical Wnt signaling is present. If the canonical Wnt signal discontinues, differentiation is directed to some other stable regulatory state. Thus, robustness of a stable regulatory state is required within the network, but also needs to be disabled by defined signals in order to assure directed development.

### Suitability of the Choosen Model to Simulate Cardiac Development

Commonly, time dependent processes are modeled with differential equations, which relate the change of a compound to its value. These models describe networks of genes and require the knowledge of concentrations for each involved species and kinetic rates for each specified interaction [33], [34], [35]. Changes in time and space further require additional parameters and can be modeled using partial differential equations [36], [37] or stochastic approaches for low molecule numbers [38]. Differential equations and stochastic approaches can accurately represent biological systems if the quantitative data is available, which is rarely the case for large systems [39]. In contrast, many approaches for modeling gene regulatory networks are based on Boolean networks. Boolean models were used for describing the cell cycle [40] or regulation of segment polarity genes in Drosophila melanogaster [41]. Even small Boolean networks with only a few genes show dynamic behavior, which resembles experiments [42]. Another example for a Boolean representation is the lac operon [18]. It demonstrates activation and repression of gene transcription with bistability by Boolean logic. These examples for Boolean models of gene regulatory networks allow for a qualitative investigation and are suitable for the analysis of larger networks [43], [44].

The specified Boolean model represents states of single entities by two values. This is a simplification of gene expression since *in vivo* all factors can occur in more than two concentration levels. The Boolean model is supported by the available data as in many expression profiles discrete values are used describing either the presence or absence of a transcription factor. The presence of factors in embryonic tissue is mostly assessed by in-situ hybridisations or immunostainings and thereby provides qualitative data. Additionally, the dependencies in the expression of factors are often given just as positive or negative regulations. Some interactions are measured by RT-PCR and stainings. This qualitative data on states and relations can be translated without many further considerations or interpretations into Boolean values and functions. Nevertheless, the model integrates the different findings on the cardiac gene regulatory network found in the literature without inconsistencies. All input data used to model the Boolean network is solely derived from literature. No estimations of mechanisms and kinetic parameters or concentrations from the available data were needed which would have been required for a more complex model such as one based on differential equations. The cardiac network model fulfills all conditions, which were derived from various experimental studies found. Based on these temporal and spatial expression and interaction data, the network model attains a high validity. In order to fully validate the network model, the expression pattern for all genes at all temporal stages in the specific subset of cells would need to be determined. Furthermore, knock-out or overexpression of these factors and non cardiac signals in the respective developmental area would help to test defined knock-out or overexpression situations of the cardiac regulatory network. These data compared to the simulation of the Boolean network model would in the end fully clarify whether the model completely displays and predicts developmental processes or not. Only a limited amount of such experimental data is available. Known results have been compared to the results of the simulated Boolean model of the core cardiac gene regulatory network and confirm our model.

### Future Extensions of the Boolean Model

Beyond the regulations presented in the network, many more factors, which are known to be expressed during cardiac development, were not yet integrated into the model. Including these factors and further interactions might refine the current model. As new data on these relations become available the computational model of the cardiac gene regulatory network will be expanded to incorporate the new knowledge. This data might be derived by multiple methods. These include time series of gene expression data in cardiac tissue, prediction of transcription factor binding sites in regulatory regions of cardiac genes, monitoring whole genome transcription factor binding sites of factors of interest by chromatin IP as well as by gene expression profiling upon loss or gain of function of cardiac specific transcription factors in geneticaly modified mice or differentiating murine ES cells. Recently developed tools that allow for binarization of gene expression data [45] and reverse engineering approaches [46], [47] will be of relevance for these purposes. Furthermore, logical models using more than two states can provide an extension to Boolean models [48], [49]. In these models intermediate values for gene states are needed requiring appropriate biological observations [50]. These and other extensions, like stochastic updates, spatial dependency, or the restriction to certain subclasses of models can then bridge the gaps as more data becomes available.

In summary, our here established initial model of the gene regulatory network covering early cardiac development fulfills all specified expectations and reproduces temporal and spatial gene expression in early murine cardiogenesis. Thereby it helps to deepen the picture of gene regulation dynamics during early cardiogenesis including the consequences of misregulation as is shown by the knock-out simulations. Furthermore, this Boolean model will be the foundation for a growing gene regulation model and further targeted experiments.

## Materials and Methods

To generate the cardiac gene regulatory network, we collected literature data about temporal and spatial expression of key genes regulating cardiogenesis and their regulatory relations. These were gained from experimental studies in mice, murine ES cells and in two cases in a human cell line. A core set of genes was selected for implementation as a Boolean model. Network components of the model were chosen by their significance for heart development. This is given by an expression between embryonic day 6.0 and 8.5 of heart development in mouse, and a sufficient amount of data available concerning the regulation of expression. To concentrate on core regulatory effects, genes integrated into the network contain input as well as output relations. Furthermore, the functional importance of genes involved has been shown by impairment of cardiogenesis upon their knock-out in mice. Network figures were drawn with Biotapestry (www.biotapestry.org). Simulations of the Boolean network were performed with the R package BoolNet [51] in R (www.r-project.org).

Our model contains four external signals (non cardiac BMP2 (exogen BMP2 I+II), non cardiac CanWnt (exogen CanWnt I+II). They do not correspond to genes within the cardiac gene regulatory network, but represent regulation input from non-cardiac tissues as this has been described for cardiogenic cells. The exogen BMP2 I and exogen BMP2 II represent a Bmp2 signal from the endoderm while exogen CanWnt I and exogen CanWnt II are required to reactivate cardiac canonical Wnt signaling in the SHF at E7.5. Both external signals are represented by two non-cardiac inputs in order to convey a temporal delay of two time steps of the non-cardiac signals into the Boolean model of the cardiac gene regulatory network.

## Supporting Information

Table S1 **Regulations of cardiac factors as depicted in** **Figure 1** **and their literature references.**
(DOC)

Table S2 **Regulatory interactions used in the cardiac regulatory network model.**
(PDF)

Table S3 **Spatial and temporal expression pattern of cardiac factors involved in the computational cardiac network model.**
(DOC)
