# Supplementary material for: A Boolean Model of the Cardiac Gene Regulatory Network Determining First and Second Heart Field Identity
Source: PLoS One. 2012 Oct 2;7(10):e46798. doi: 10.1371/journal.pone.0046798 (PMC3462786; doi:10.1371/journal.pone.0046798)
Supplement: Table S1 — Regulations of cardiac factors as depicted in Figure 1 and their literature references. (DOC) [file pone.0046798.s001.doc]

# Supplementary table 1: Regulations of cardiac factors as depicted in figure 1 and their literature references.

| factor | regulates | target | reference | comment |
| --- | --- | --- | --- | --- |
| canWnt | up | Mesp1 | [1] |  |
| canWnt | up | T | [2,3] |  |
| canWnt | up | Gsc | [3] |  |
| canWnt | up | Bmp2 | [4] |  |
| canWnt | up | Wnt11 | [5] |  |
| canWnt | up | Fgf10 | [6] |  |
| canWnt | up | Isl1 | [7,8] |  |
| canWnt | up | Bmp4 | [8] |  |
| Dkk1 | down | Tbx5 | [9] | without Mesp1 |
| Dkk1 | down | Tbx20 | [9] | without Mesp1 |
| Dkk1 | down | Hand1 | [9] | without Mesp1 |
| Dkk1 | down | Hand2 | [9] | without Mesp1 |
| Dkk1 | up | Nkx2.5 | [9] | with Mesp1 |
| Fgf8 | up | Isl1 | [10,11] |  |
| Foxa2 | up | Tbx1 | [12] |  |
| Foxc1/2 | up | Tbx1 | [12,13] |  |
| Foxh1 | up | Fgf8 | [14] |  |
| Foxh1 | up | Fgf10 | [14] |  |
| Foxh1 | up | Mef2c | [14] |  |
| GATA4 | up | Nkx2.5 | [15,16] |  |
| GATA4 | up | Bmp4 | [17] |  |
| GATA4 | up | Mef2c | [16,18] |  |
| GATA4 | up | Hand2 | [19] |  |
| GATA4 | up | aMHC | [20] |  |
| GATA4 | up | bMHC | [21] |  |
| GATA4 | up | Nppa | [22] |  |
| Hand1 | up | Nppa | [23] |  |
| Hand2 | up | Irx4 | [24] |  |
| Hand2 | up | Nppa | [23] |  |
| Isl1 | up | Bmp4 | [25] |  |
| Isl1 | up | Nkx2.5 | [16] |  |
| Isl1 | up | Fgf10 | [25] |  |
| Isl1 | up | Mef2c | [16,18,26] |  |
| Isl1 | up | Shh | [27] |  |
| Irx4 | up | Hand1 | [28] |  |
| Mef2c | up | Hand2 | [29] |  |
| Mef2c | down | Hand1 | [29] |  |
| Mef2c | up | aMHC | [29] |  |
| Mef2c | up | aActin | [29] |  |
| Mef2c | up | MLC1a | [29] |  |
| Mesp1 | down | Mesp1 | [9] |  |
| Mesp1 | down | T | [1,9] |  |
| Mesp1 | down | Gsc | [1] |  |
| Mesp1 | up | Nkx2.5 | [1,9] |  |
| Mesp1 | up | Gata4 | [1,9] |  |
| Mesp1 | up | Tbx5 | [9] |  |
| Mesp1 | up | Foxh1 | [1] |  |
| Mesp1 | up | Tbx1 | [9] |  |
| Mesp1 | up | Isl1 | [9] |  |
| Mesp1 | down | Fgf8 | [1,9] |  |
| Mesp1 | up | Hand2 | [1] |  |
| Mesp1 | up | Myocardin | [1] |  |
| Myocardin | up | MLC2v | [30] |  |
| Myocardin | up | Nppa | [30] |  |
| Myocardin | up | aMHC | [30] |  |
| Nkx2.5 | down | Fgf10 | [31] |  |
| Nkx2.5 | up | Irx4 | [24] |  |
| Nkx2.5 | up | Wnt11 | [31] |  |
| Nkx2.5 | up | Mef2c | [14,16,32] | |
| Nkx2.5 | down | Bmp2 | [31] |  |
| Nkx2.5 | up | Tbx5 | [33] |  |
| Nkx2.5 | up | Hand1 | [32] |  |
| Nkx2.5 | up | Myocardin | [34] |  |
| Nkx2.5 | up | Nppa | [32,35] |  |
| Shh | up | Foxa2 | [36] |  |
| Shh | up | Foxc1/2 | [36] |  |
| Bmp2/Smad1/5/8 | up | Nkx2.5 | [15,37] |  |
| Bmp2/Smad1/5/8 | up | Tbx2 | [38] |  |
| Tbx1 | up | Foxa2 | [39] |  |
| Tbx1 | up | Fgf8 | [39] |  |
| Tbx1 | up | Fgf10 | [16] |  |
| Tbx1 | up | Nkx2.5 | [16] |  |
| Tbx1 | up | Isl1 | [40] |  |
| Tbx1 | down | GATA4 | [40] |  |
| Tbx1 | down | Tbx5 | [40] |  |
| Tbx2 | down | Nppa | [41] |  |
| Tbx5 | up | Nkx2.5 | [33,35,42] |  |
| Tbx5 | up | GATA4 | [43] |  |
| Tbx5 | up | Tbx5 | [33,42] |  |
| Tbx5 | up | Mef2c | [35,42] |  |
| Tbx5 | up | Nppa | [42,44] |  |
| Tbx5 | up | aMHC | [45] |  |
| Tbx20 | up | Mef2c | [16] |  |
| Tbx20 | up | Nkx2.5 | [16] |  |
| Tbx20 | down | Tbx2 | [46] |  |
| Tbx20 | down | Bmp2 | [47] |  |
| Tbx20 | down | Tbx5 | [47] |  |

References

1. Bondue A, Lapouge G, Paulissen C, Semeraro C, Iacovino M, et al. (2008) Mesp1 acts as a master regulator of multipotent cardiovascular progenitor specification. Cell stem cell 3: 69-84.

2. Arnold SJ, Stappert J, Bauer A, Kispert A, Herrmann BG, et al. (2000) Brachyury is a target gene of the Wnt/beta-catenin signaling pathway. Mechanisms of development 91: 249-258.

3. Liu P, Wakamiya M, Shea MJ, Albrecht U, Behringer RR, et al. (1999) Requirement for Wnt3 in vertebrate axis formation. Nature genetics 22: 361-365.

4. Nakamura T, Sano M, Songyang Z, Schneider MD (2003) A Wnt- and beta -catenin-dependent pathway for mammalian cardiac myogenesis. Proceedings of the National Academy of Sciences of the United States of America 100: 5834-5839.

5. Ueno S, Weidinger G, Osugi T, Kohn AD, Golob JL, et al. (2007) Biphasic role for Wnt/beta-catenin signaling in cardiac specification in zebrafish and embryonic stem cells. Proceedings of the National Academy of Sciences of the United States of America 104: 9685-9690.

6. Cohen ED, Wang Z, Lepore JJ, Lu MM, Taketo MM, et al. (2007) Wnt/beta-catenin signaling promotes expansion of Isl-1-positive cardiac progenitor cells through regulation of FGF signaling. J Clin Invest 117: 1794-1804.

7. Lin L, Cui L, Zhou W, Dufort D, Zhang X, et al. (2007) Beta-catenin directly regulates Islet1 expression in cardiovascular progenitors and is required for multiple aspects of cardiogenesis. Proceedings of the National Academy of Sciences of the United States of America 104: 9313-9318.

8. Klaus A, Saga Y, Taketo MM, Tzahor E, Birchmeier W (2007) Distinct roles of Wnt/beta-catenin and Bmp signaling during early cardiogenesis. Proceedings of the National Academy of Sciences of the United States of America 104: 18531-18536.

9. Lindsley RC, Gill JG, Murphy TL, Langer EM, Cai M, et al. (2008) Mesp1 coordinately regulates cardiovascular fate restriction and epithelial-mesenchymal transition in differentiating ESCs. Cell stem cell 3: 55-68.

10. Ilagan R, Abu-Issa R, Brown D, Yang YP, Jiao K, et al. (2006) Fgf8 is required for anterior heart field development. Development (Cambridge, England) 133: 2435-2445.

11. Park EJ, Ogden LA, Talbot A, Evans S, Cai CL, et al. (2006) Required, tissue-specific roles for Fgf8 in outflow tract formation and remodeling. Development (Cambridge, England) 133: 2419-2433.

12. Maeda J, Yamagishi H, McAnally J, Yamagishi C, Srivastava D (2006) Tbx1 is regulated by forkhead proteins in the secondary heart field. Developmental dynamics : an official publication of the American Association of Anatomists 235: 701-710.

13. Seo S, Kume T (2006) Forkhead transcription factors, Foxc1 and Foxc2, are required for the morphogenesis of the cardiac outflow tract. Developmental biology 296: 421-436.

14. von Both I, Silvestri C, Erdemir T, Lickert H, Walls JR, et al. (2004) Foxh1 is essential for development of the anterior heart field. Developmental cell 7: 331-345.

15. Brown CO, 3rd, Chi X, Garcia-Gras E, Shirai M, Feng XH, et al. (2004) The cardiac determination factor, Nkx2-5, is activated by mutual cofactors GATA-4 and Smad1/4 via a novel upstream enhancer. The Journal of biological chemistry 279: 10659-10669.

16. Takeuchi JK, Mileikovskaia M, Koshiba-Takeuchi K, Heidt AB, Mori AD, et al. (2005) Tbx20 dose-dependently regulates transcription factor networks required for mouse heart and motoneuron development. Development (Cambridge, England) 132: 2463-2474.

17. Nemer G, Nemer M (2003) Transcriptional activation of BMP-4 and regulation of mammalian organogenesis by GATA-4 and -6. Developmental biology 254: 131-148.

18. Dodou E, Verzi MP, Anderson JP, Xu SM, Black BL (2004) Mef2c is a direct transcriptional target of ISL1 and GATA factors in the anterior heart field during mouse embryonic development. Development (Cambridge, England) 131: 3931-3942.

19. Zhao R, Watt AJ, Battle MA, Li J, Bondow BJ, et al. (2008) Loss of both GATA4 and GATA6 blocks cardiac myocyte differentiation and results in acardia in mice. Dev Biol 317: 614-619.

20. Molkentin JD, Kalvakolanu DV, Markham BE (1994) Transcription Factor GATA-4 Regulates Cardiac Muscle-Specific Expression of the a-Myosin Heavy-Chain Gene. Molecular & Cellular Biology 14: 4947-4957.

21. Charron F, Paradis P, Bronchain O, Nemer G, Nemer M (1999) Cooperative interaction between GATA-4 and GATA-6 regulates myocardial gene expression. Mol Cell Biol 19: 4355-4365.

22. Temsah R, Nemer M (2005) GATA factors and transcriptional regulation of cardiac natriuretic peptide genes. Regul Pept 128: 177-185.

23. Morin S, Pozzulo G, Robitaille L, Cross J, Nemer M (2005) MEF2-dependent recruitment of the HAND1 transcription factor results in synergistic activation of target promoters. J Biol Chem 280: 32272-32278.

24. Bruneau BG, Bao ZZ, Tanaka M, Schott JJ, Izumo S, et al. (2000) Cardiac expression of the ventricle-specific homeobox gene Irx4 is modulated by Nkx2-5 and dHand. Developmental biology 217: 266-277.

25. Cai CL, Liang X, Shi Y, Chu PH, Pfaff SL, et al. (2003) Isl1 identifies a cardiac progenitor population that proliferates prior to differentiation and contributes a majority of cells to the heart. Developmental cell 5: 877-889.

26. Black BL (2007) Transcriptional pathways in second heart field development. Semin Cell Dev Biol 18: 67-76.

27. Lin L, Bu L, Cai CL, Zhang X, Evans S (2006) Isl1 is upstream of sonic hedgehog in a pathway required for cardiac morphogenesis. Developmental biology 295: 756-763.

28. Bruneau BG, Bao ZZ, Fatkin D, Xavier-Neto J, Georgakopoulos D, et al. (2001) Cardiomyopathy in Irx4-deficient mice is preceded by abnormal ventricular gene expression. Molecular and cellular biology 21: 1730-1736.

29. Lin Q, Schwarz J, Bucana C, Olson EN (1997) Control of mouse cardiac morphogenesis and myogenesis by transcription factor MEF2C. Science (New York, NY) 276: 1404-1407.

30. Wang C, Cao D, Wang Q, Wang DZ (2001) Synergistic activation of cardiac genes by myocardin and Tbx5. PLoS One 6: e24242.

31. Prall OW, Menon MK, Solloway MJ, Watanabe Y, Zaffran S, et al. (2007) An Nkx2-5/Bmp2/Smad1 negative feedback loop controls heart progenitor specification and proliferation. Cell 128: 947-959.

32. Tanaka M, Wechsler SB, Lee IW, Yamasaki N, Lawitts JA, et al. (1999) Complex modular cis-acting elements regulate expression of the cardiac specifying homeobox gene Csx/Nkx2.5. Development 126: 1439-1450.

33. Sun G, Lewis LE, Huang X, Nguyen Q, Price C, et al. (2004) TBX5, a gene mutated in Holt-Oram syndrome, is regulated through a GC box and T-box binding elements (TBEs). Journal of cellular biochemistry 92: 189-199.

34. Ueyama T, Kasahara H, Ishiwata T, Nie Q, Izumo S (2003) Myocardin expression is regulated by Nkx2.5, and its function is required for cardiomyogenesis. Molecular and cellular biology 23: 9222-9232.

35. Hiroi Y, Kudoh S, Monzen K, Ikeda Y, Yazaki Y, et al. (2001) Tbx5 associates with Nkx2-5 and synergistically promotes cardiomyocyte differentiation. Nature genetics 28: 276-280.

36. Yamagishi H, Maeda J, Hu T, McAnally J, Conway SJ, et al. (2003) Tbx1 is regulated by tissue-specific forkhead proteins through a common Sonic hedgehog-responsive enhancer. Genes & development 17: 269-281.

37. Liberatore CM, Searcy-Schrick RD, Vincent EB, Yutzey KE (2002) Nkx-2.5 gene induction in mice is mediated by a Smad consensus regulatory region. Developmental biology 244: 243-256.

38. Yamada M, Revelli JP, Eichele G, Barron M, Schwartz RJ (2000) Expression of chick Tbx-2, Tbx-3, and Tbx-5 genes during early heart development: evidence for BMP2 induction of Tbx2. Dev Biol 228: 95-105.

39. Hu T, Yamagishi H, Maeda J, McAnally J, Yamagishi C, et al. (2004) Tbx1 regulates fibroblast growth factors in the anterior heart field through a reinforcing autoregulatory loop involving forkhead transcription factors. Development (Cambridge, England) 131: 5491-5502.

40. Liao J, Aggarwal VS, Nowotschin S, Bondarev A, Lipner S, et al. (2008) Identification of downstream genetic pathways of Tbx1 in the second heart field. Developmental biology 316: 524-537.

41. Habets PE, Moorman AF, Clout DE, van Roon MA, Lingbeek M, et al. (2002) Cooperative action of Tbx2 and Nkx2.5 inhibits ANF expression in the atrioventricular canal: implications for cardiac chamber formation. Genes Dev 16: 1234-1246.

42. Herrmann F, Bundschu K, Kuhl SJ, Kuhl M Tbx5 overexpression favors a first heart field lineage in murine embryonic stem cells and in Xenopus laevis embryos. Dev Dyn 240: 2634-2645.

43. Bruneau BG, Nemer G, Schmitt JP, Charron F, Robitaille L, et al. (2001) A murine model of Holt-Oram syndrome defines roles of the T-box transcription factor Tbx5 in cardiogenesis and disease. Cell 106: 709-721.

44. Mori AD, Zhu Y, Vahora I, Nieman B, Koshiba-Takeuchi K, et al. (2006) Tbx5-dependent rheostatic control of cardiac gene expression and morphogenesis. Dev Biol 297: 566-586.

45. Wang C, Cao D, Wang Q, Wang DZ (2011) Synergistic activation of cardiac genes by myocardin and Tbx5. PLoS One 6: e24242.

46. Singh MK, Christoffels VM, Dias JM, Trowe MO, Petry M, et al. (2005) Tbx20 is essential for cardiac chamber differentiation and repression of Tbx2. Development (Cambridge, England) 132: 2697-2707.

47. Stennard FA, Costa MW, Lai D, Biben C, Furtado MB, et al. (2005) Murine T-box transcription factor Tbx20 acts as a repressor during heart development, and is essential for adult heart integrity, function and adaptation. Development (Cambridge, England) 132: 2451-2462.
