# Supplementary material for: A Boolean Model of the Cardiac Gene Regulatory Network Determining First and Second Heart Field Identity
Source: PLoS One. 2012 Oct 2;7(10):e46798. doi: 10.1371/journal.pone.0046798 (PMC3462786; doi:10.1371/journal.pone.0046798)
Supplement: Table S2 — Regulatory interactions used in the cardiac regulatory network model. (PDF) [file pone.0046798.s002.pdf]

Supplementary table 2: **Regulatory interactions used in the cardiac regulatory network model**

factor	regulates	target	reference	model system	stage	method	comment
canWnt	down	Bmp2	1	ES cells		RT-PCR	
canWnt	up	Dkk1	2	ES cells		qPCR	Wnt3a
Mesp1	up	Dkk1	3	mouse, ES cells		transgenic mouse, ChIP, EMSA, qPCR, luciferase reporter assay	human Dkk1
Foxc1/2	up	Fgf8	4	mouse, SHF	E8.5	transgenic mouse	
Mesp1	down	Fgf8	5	ES cells		RT-PCR, microarray	
Tbx1	up	Fgf8	6	mouse, AHF, OFT	E9.5	transgenic mouse	
canWnt	up	Foxc1/2	7;8	ES cells, P19 cells		qPCR, microarray	skeletal muscle development
Mesp1	up	GATA	9;3;5	ES cells		ChIP, RT-PCR, qPCR, microarray	GATA4
Nkx2.5	up	GATA	10	10T1/2 cells		promoter studies, EMSA	GATA6
Tbx5	up	GATA	11	mouse	E8.0 - E8.5	transgenic mouse	
canWnt	up	Isl1	12;13	mouse, SHF	E8 - E10	transgenic mouse, ChIP, luciferase reporter assay	
Fgf8	up	Isl1	14	mouse	7-11ss	transgenic mouse, Immunostaining	
Mesp1	up	Isl1	5;9	ES cells		RT-PCR, Immunostaining	
Tbx1	up	Isl1	15	mouse, SHF	E8.75	transgenic mouse, qPCR, microarray	
Bmp2	up	Nkx2.5	16;17	mouse, P19 cells	from E7.5	transgenic mouse, reporter assay, ChIP	Smads tested; together with GATAs
canWnt	up	Mesp1	2	ES cells		qPCR	
GATA	up	Nkx2.5	16	mouse, P19 cells	from E7.5	transgenic mouse, reporter assay, EMSA, ChIP	together with Smads
Isl1	up	Nkx2.5	18	mouse, 10T1/2 cells	E9.75	transgenic mouse, luciferase reporter assay	together with GATAs
Mesp1	up	Nkx2.5	9;5;3	ES cells		RT-PCR, microarray, Immunostaining	Mesp1 and Dkk1
Tbx1	up	Nkx2.5	18	10T1/2 cells		luciferase reporter assay	
Tbx5	up	Nkx2.5	18;11	mouse, 10T1/2 cells	E8.5, E10	transgenic mouse, luciferase reporter assay	
Foxc1/2	up	Tbx1	4;19	mouse, SHF, COS cells	E8.5, E9.5	transgenic mouse, EMSA, luciferase reporter assay	
canWnt	down	Tbx5	1	ES cells	later stage	RT-PCR	
Dkk1	down	Tbx5	9	ES cells		RT-PCR, microarray	if Mesp1 is not present
Mesp1	up	Tbx5	9	ES cells		RT-PCR, microarray	
Nkx2.5	up	Tbx5	20	human cells		footprint assay, EMSA	
Tbx1	down	Tbx5	15	mouse, SHF	E8.75 - E10.75	transgenic mouse, microarray, qPCR	
Tbx5	up	Tbx5	20	human cells		footprint assay, EMSA	

References

[1] Naito AT, Shiojima I, Akazawa H, Hidaka K, Morisaki T, et al. (2006) Developmental stage-specific biphasic roles of wnt/beta-catenin signaling in cardiomyogenesis and hematopoiesis. Proceedings of the National Academy of Sciences of the United States of America 103: 19812-19817.

[2] Ueno S, Weidinger G, Osugi T, Kohn AD, Golob JL, et al. (2007) Biphasic role for wnt/beta-catenin signaling in cardiac specification in zebrafish and embryonic stem cells. Proceedings of the National Academy of Sciences of the United States of America 104: 9685-9690.

[3] David R, Brenner C, Stieber J, Schwarz F, Brunner S, et al. (2008) Mesp1 drives vertebrate cardiovascular differentiation through dkk-1-mediated blockade of wnt-signalling. Nature cell biology 10: 338-345.

[4] Seo S, Kume T (2006) Forkhead transcription factors, foxc1 and foxc2, are required for the morphogenesis of the cardiac outflow tract. Developmental biology 296: 421-436.

[5] Bondue A, Lapouge G, Paulissen C, Semeraro C, Iacovino M, et al. (2008) Mesp1 acts as a master regulator of multipotent cardiovascular progenitor specification. Cell stem cell 3: 69-84.

[6] Hu T, Yamagishi H, Maeda J, McAnally J, Yamagishi C, et al. (2004) Tbx1 regulates fibroblast growth factors in the anterior heart field through a reinforcing autoregulatory loop involving forkhead transcription factors. Development (Cambridge, England) 131: 5491-5502.

[7] Liu Y, Asakura M, Inoue H, Nakamura T, Sano M, et al. (2007) Sox17 is essential for the specification of cardiac mesoderm in embryonic stem cells. Proceedings of the National Academy of Sciences of the United States of America 104: 3859-3864.

[8] Savage J, Voronova A, Mehta V, Sendi-Mukasa F, Skerjanc IS (2010) Canonical wnt signaling regulates foxc1/2 expression in p19 cells. Differentiation; research in biological diversity 79: 31-40.

[9] Lindsley RC, Gill JG, Murphy TL, Langer EM, Cai M, et al. (2008) Mesp1 coordinately regulates cardiovascular fate restriction and epithelial-mesenchymal transition in differentiating escs. Cell stem cell 3: 55-68.

[10] Molkentin JD, Antos C, Mercer B, Taigen T, Miano JM, et al. (2000) Direct activation of a gata6 cardiac enhancer by nkx2.5: evidence for a reinforcing regulatory network of nkx2.5 and gata transcription factors in the developing heart. Developmental biology 217: 301-309.

[11] Bruneau BG, Nemer G, Schmitt JP, Charron F, Robitaille L, et al. (2001) A murine model of holt-oram syndrome defines roles of the t-box transcription factor tbx5 in cardiogenesis and disease. Cell 106: 709-721.

[12] Klaus A, Saga Y, Taketo MM, Tzahor E, Birchmeier W (2007) Distinct roles of wnt/beta-catenin and bmp signaling during early cardiogenesis. Proceedings of the National Academy of Sciences of the United States of America 104: 18531-18536.

[13] Lin L, Cui L, Zhou W, Dufort D, Zhang X, et al. (2007) Beta-catenin directly regulates islet1 expression in cardiovascular progenitors and is required for multiple aspects of cardiogenesis. Proceedings of the National Academy of Sciences of the United States of America 104: 9313-9318.

[14] Park EJ, Ogden LA, Talbot A, Evans S, Cai CL, et al. (2006) Required, tissue-specific roles for fgf8 in outflow tract formation and remodeling. Development (Cambridge, England) 133: 2419-2433.

[15] Liao J, Aggarwal VS, Nowotschin S, Bondarev A, Lipner S, et al. (2008) Identification of downstream genetic pathways of tbx1 in the second heart field. Developmental biology 316: 524-537.

[16] 3rd COB, Chi X, Garcia-Gras E, Shirai M, Feng XH, et al. (2004) The cardiac determination factor, nkx2-5, is activated by mutual cofactors gata-4 and smad1/4 via a novel upstream enhancer. The Journal of biological chemistry 279: 10659-10669.

[17] Liberatore CM, Searcy-Schrick RD, Vincent EB, Yutzey KE (2002) Nkx-2.5 gene induction in mice is mediated by a smad consensus regulatory region. Developmental biology 244: 243-256.

[18] Takeuchi JK, Mileikovskaia M, Koshiba-Takeuchi K, Heidt AB, Mori AD, et al. (2005) Tbx20 dose-dependently regulates transcription factor networks required for mouse heart and motoneuron development. Development (Cambridge, England) 132: 2463-2474.

[19] Maeda J, Yamagishi H, McAnally J, Yamagishi C, Srivastava D (2006) Tbx1 is regulated by forkhead proteins in the secondary heart field. Developmental dynamics : an official publication of the American Association of Anatomists 235: 701-710.

[20] Sun G, Lewis LE, Huang X, Nguyen Q, Price C, et al. (2004) Tbx5, a gene mutated in holt-oram syndrome, is regulated through a gc box and t-box binding elements (tbes). Journal of cellular biochemistry 92: 189-199.
