# Supplementary material for: A Boolean Model of the Cardiac Gene Regulatory Network Determining First and Second Heart Field Identity
Source: PLoS One. 2012 Oct 2;7(10):e46798. doi: 10.1371/journal.pone.0046798 (PMC3462786; doi:10.1371/journal.pone.0046798)
Supplement: Table S3 — Spatial and temporal expression pattern of cardiac factors involved in the computational cardiac network model. (DOC) [file pone.0046798.s003.doc]

| factor | temporal expression  between gastrulation  and E8.5 | spatial expression | reference |
| --- | --- | --- | --- |
| Bmp2 | E7.5 –E8.5 | cardiac crescent; sinus venosus; unclear whether expression is in FHF, SHF or endoderm; is able to signal to precardiac mesoderm | [1,2,3] |
| canonical  Wnts (early)  canonical  Wnts  (late) | E6.0 – E6.5  E7.5 | Wnt3 in the primitive streak  SHF | [4,5]  [6,7,8,9,10] |
| Dkk1 | E7.5, E8.5 | crescent shape in anterior endoderm, foregut endoderm | [11,12] |
| Fgf8 | E7.75 – E8.5 | SHF and signals to the AHF | [13,14] |
| Foxc1/2 | ab E7.75 | SHF | [13,15] |
| GATAs | from E7.0/E7.5 – E8.5 | precardiac mesoderm, endoderm next to it, heart tube and also SHF | [16,17,18,19] |
| Islet-1 | E7.5 – E8.5 | SHF | [20,21] |
| Mesp1 | gastrulation, downregulated at E7.5/E8.0 | heart precursor cells of FHF and SHF | [12,13,22,23,24] |
| Nkx2.5 | from E7.5 on | FHF and SHF | [25,26,27] |
| SHH | E8.5 | endoderm; starts signaling to lateral plate mesoderm at E8.5 | [28,29,30,31,32] |
| Tbx1 | from E7.5 | SHF | [33,34] |
| Tbx5 | from E7.5 | FHF; later on in anteroposterior gradient | [8,35,36,37,38,39,40] |

**Supplementary table 3:** Spatial and temporal expression pattern of cardiac factors involved in the computational cardiac network model.

References

1. Biben C, Stanley E, Fabri L, Kotecha S, Rhinn M, et al. (1998) Murine cerberus homologue mCer-1: a candidate anterior patterning molecule. Developmental biology 194: 135-151.

2. Prall OW, Menon MK, Solloway MJ, Watanabe Y, Zaffran S, et al. (2007) An Nkx2-5/Bmp2/Smad1 negative feedback loop controls heart progenitor specification and proliferation. Cell 128: 947-959.

3. Ma L, Lu MF, Schwartz RJ, Martin JF (2005) Bmp2 is essential for cardiac cushion epithelial-mesenchymal transition and myocardial patterning. Development 132: 5601-5611.

4. Liu P, Wakamiya M, Shea MJ, Albrecht U, Behringer RR, et al. (1999) Requirement for Wnt3 in vertebrate axis formation. Nature genetics 22: 361-365.

5. Morkel M, Huelsken J, Wakamiya M, Ding J, van de Wetering M, et al. (2003) Beta-catenin regulates Cripto- and Wnt3-dependent gene expression programs in mouse axis and mesoderm formation. Development (Cambridge, England) 130: 6283-6294.

6. Klaus A, Saga Y, Taketo MM, Tzahor E, Birchmeier W (2007) Distinct roles of Wnt/beta-catenin and Bmp signaling during early cardiogenesis. Proceedings of the National Academy of Sciences of the United States of America 104: 18531-18536.

7. Kwon C, Arnold J, Hsiao EC, Taketo MM, Conklin BR, et al. (2007) Canonical Wnt signaling is a positive regulator of mammalian cardiac progenitors. Proceedings of the National Academy of Sciences of the United States of America 104: 10894-10899.

8. Ai D, Fu X, Wang J, Lu MF, Chen L, et al. (2007) Canonical Wnt signaling functions in second heart field to promote right ventricular growth. Proceedings of the National Academy of Sciences of the United States of America 104: 9319-9324.

9. Guo Q, Li JY (2007) Distinct functions of the major Fgf8 spliceform, Fgf8b, before and during mouse gastrulation. Development (Cambridge, England) 134: 2251-2260.

10. Takada S, Stark KL, Shea MJ, Vassileva G, McMahon JA, et al. (1994) Wnt-3a regulates somite and tailbud formation in the mouse embryo. Genes & development 8: 174-189.

11. Lewis SL, Khoo PL, Andrea De Young R, Bildsoe H, Wakamiya M, et al. (2007) Genetic interaction of Gsc and Dkk1 in head morphogenesis of the mouse. Mechanisms of development 124: 157-165.

12. David R, Brenner C, Stieber J, Schwarz F, Brunner S, et al. (2008) MesP1 drives vertebrate cardiovascular differentiation through Dkk-1-mediated blockade of Wnt-signalling. Nature cell biology 10: 338-345.

13. Park EJ, Ogden LA, Talbot A, Evans S, Cai CL, et al. (2006) Required, tissue-specific roles for Fgf8 in outflow tract formation and remodeling. Development (Cambridge, England) 133: 2419-2433.

14. Ilagan R, Abu-Issa R, Brown D, Yang YP, Jiao K, et al. (2006) Fgf8 is required for anterior heart field development. Development (Cambridge, England) 133: 2435-2445.

15. Seo S, Kume T (2006) Forkhead transcription factors, Foxc1 and Foxc2, are required for the morphogenesis of the cardiac outflow tract. Developmental biology 296: 421-436.

16. Heikinheimo M, Scandrett JM, Wilson DB (1994) Localization of transcription factor GATA-4 to regions of the mouse embryo involved in cardiac development. Developmental biology 164: 361-373.

17. Buckingham M, Meilhac S, Zaffran S (2005) Building the mammalian heart from two sources of myocardial cells. Nature reviewsGenetics 6: 826-835.

18. Nemer G, Nemer M (2003) Transcriptional activation of BMP-4 and regulation of mammalian organogenesis by GATA-4 and -6. Developmental biology 254: 131-148.

19. Roebroek AJ, Umans L, Pauli IG, Robertson EJ, van Leuven F, et al. (1998) Failure of ventral closure and axial rotation in embryos lacking the proprotein convertase Furin. Development (Cambridge, England) 125: 4863-4876.

20. Cai CL, Liang X, Shi Y, Chu PH, Pfaff SL, et al. (2003) Isl1 identifies a cardiac progenitor population that proliferates prior to differentiation and contributes a majority of cells to the heart. Developmental cell 5: 877-889.

21. von Both I, Silvestri C, Erdemir T, Lickert H, Walls JR, et al. (2004) Foxh1 is essential for development of the anterior heart field. Developmental cell 7: 331-345.

22. Saga Y, Kitajima S, Miyagawa-Tomita S (2000) Mesp1 expression is the earliest sign of cardiovascular development. Trends in cardiovascular medicine 10: 345-352.

23. Saga Y, Miyagawa-Tomita S, Takagi A, Kitajima S, Miyazaki J, et al. (1999) MesP1 is expressed in the heart precursor cells and required for the formation of a single heart tube. Development (Cambridge, England) 126: 3437-3447.

24. Kitajima S, Takagi A, Inoue T, Saga Y (2000) MesP1 and MesP2 are essential for the development of cardiac mesoderm. Development (Cambridge, England) 127: 3215-3226.

25. Searcy RD, Vincent EB, Liberatore CM, Yutzey KE (1998) A GATA-dependent nkx-2.5 regulatory element activates early cardiac gene expression in transgenic mice. Development (Cambridge, England) 125: 4461-4470.

26. Stanley EG, Biben C, Elefanty A, Barnett L, Koentgen F, et al. (2002) Efficient Cre-mediated deletion in cardiac progenitor cells conferred by a 3'UTR-ires-Cre allele of the homeobox gene Nkx2-5. The International journal of developmental biology 46: 431-439.

27. Komuro I, Izumo S (1993) Csx: a murine homeobox-containing gene specifically expressed in the developing heart. Proceedings of the National Academy of Sciences of the United States of America 90: 8145-8149.

28. Zaffran S, Frasch M (2002) Early signals in cardiac development. Circulation research 91: 457-469.

29. Zhang XM, Ramalho-Santos M, McMahon AP (2001) Smoothened mutants reveal redundant roles for Shh and Ihh signaling including regulation of L/R symmetry by the mouse node. Cell 106: 781-792.

30. Warr N, Powles-Glover N, Chappell A, Robson J, Norris D, et al. (2008) Zic2-associated holoprosencephaly is caused by a transient defect in the organizer region during gastrulation. Human molecular genetics 17: 2986-2996.

31. Bai CB, Auerbach W, Lee JS, Stephen D, Joyner AL (2002) Gli2, but not Gli1, is required for initial Shh signaling and ectopic activation of the Shh pathway. Development (Cambridge, England) 129: 4753-4761.

32. Lin L, Bu L, Cai CL, Zhang X, Evans S (2006) Isl1 is upstream of sonic hedgehog in a pathway required for cardiac morphogenesis. Developmental biology 295: 756-763.

33. Brown CO, 3rd, Chi X, Garcia-Gras E, Shirai M, Feng XH, et al. (2004) The cardiac determination factor, Nkx2-5, is activated by mutual cofactors GATA-4 and Smad1/4 via a novel upstream enhancer. The Journal of biological chemistry 279: 10659-10669.

34. Yamagishi H, Maeda J, Hu T, McAnally J, Conway SJ, et al. (2003) Tbx1 is regulated by tissue-specific forkhead proteins through a common Sonic hedgehog-responsive enhancer. Genes & development 17: 269-281.

35. Christoffels VM, Habets PE, Franco D, Campione M, de Jong F, et al. (2000) Chamber formation and morphogenesis in the developing mammalian heart. Developmental biology 223: 266-278.

36. Bruneau BG, Nemer G, Schmitt JP, Charron F, Robitaille L, et al. (2001) A murine model of Holt-Oram syndrome defines roles of the T-box transcription factor Tbx5 in cardiogenesis and disease. Cell 106: 709-721.

37. Liberatore CM, Searcy-Schrick RD, Yutzey KE (2000) Ventricular expression of tbx5 inhibits normal heart chamber development. Developmental biology 223: 169-180.

38. Chapman DL, Garvey N, Hancock S, Alexiou M, Agulnik SI, et al. (1996) Expression of the T-box family genes, Tbx1-Tbx5, during early mouse development. Developmental dynamics : an official publication of the American Association of Anatomists 206: 379-390.

39. Zaffran S, Kelly RG, Meilhac SM, Buckingham ME, Brown NA (2004) Right ventricular myocardium derives from the anterior heart field. Circulation research 95: 261-268.

40. Hochgreb T, Linhares VL, Menezes DC, Sampaio AC, Yan CY, et al. (2003) A caudorostral wave of RALDH2 conveys anteroposterior information to the cardiac field. Development (Cambridge, England) 130: 5363-5374.
